# Arterial Blood Gases and Cardiorespiratory Parameters in Etorphine-Medetomidine-Midazolam Immobilized Free-Ranging and Game-Farmed Southern White Rhinoceroses (*Ceratotherium simum simum*) Undergoing Electro-Ejaculation

**DOI:** 10.3389/fvets.2022.862100

**Published:** 2022-04-27

**Authors:** Janine Meuffels, Henk Bertschinger, Brendan Tindall, Friederike Pohlin, Ilse Luther-Binoir, Shweta Trivedi, Christiaan R. Boshoff, Imke Lueders

**Affiliations:** ^1^Cryovault, Hemmersbach Rhino Force NCP, Hoedspruit, South Africa; ^2^Department of Production Animal Studies, Faculty of Veterinary Science, University of Pretoria, Pretoria, South Africa; ^3^Robberg Veterinary Clinic, Plettenberg Bay, South Africa; ^4^Department of Interdisciplinary Life Sciences, Research Institute of Wildlife Ecology, University of Veterinary Medicine Vienna, Vienna, Austria; ^5^GEOsperm, Wildlife Reproduction and Biotechnology Services, Brits, South Africa; ^6^Department of Animal Science, North Carolina State University, Raleigh, NC, United States; ^7^Wild Game Veterinary Services, Gravelotte, South Africa; ^8^Mammal Research Institute, Faculty of Natural and Agricultural Sciences, University of Pretoria, Hatfield, South Africa

**Keywords:** white rhinoceros (*Ceratotherium simum*), medetomidine, midazolam, arterial blood gases, blood pressure, semen collection, immobilization, cardiorespiratory variables

## Abstract

With the rapid loss of individuals in the wild, semen cryopreservation has gained importance to safeguard the genetic diversity of white rhinoceroses (*Ceratotherium simum*). For semen collection *via* electro-ejaculation, immobilization of free-ranging individuals requires the potent opioid etorphine, which is routinely combined with azaperone, but causes hypoxemia, hypercarbia, acidemia, muscle rigidity, tachycardia, and systemic hypertension. In this study, the suitability of two alternative immobilization protocols including etorphine, medetomidine, and midazolam at different doses (high vs. low etorphine) was evaluated in adult white rhinoceros bulls in two different management systems (free-ranging vs. game-farmed) and undergoing electro-ejaculation. Fourteen free-ranging (Group 1) and 28 game-farmed rhinoceroses (Group 2) were immobilized with ≈2.5 μg/kg etorphine (high dose), ≈2.5 μg/kg medetomidine, ≈25 μg/kg midazolam and 1,500–1,700 IU hyaluronidase and received ≈2.5 μg/kg of butorphanol intravenously at first handling. Twenty game-farmed animals (Group 3) received ≈1 μg/kg etorphine (low dose), ≈5 μg/kg medetomidine, ≈25 μg/kg midazolam and 1,700 IU hyaluronidase. Respiratory rate, heart rate and peripheral hemoglobin oxygen saturation (SpO_2_) were measured at 5-min intervals; non-invasive oscillometric blood pressures and arterial blood gases at first handling and before reversal of the immobilization; serum clinical chemistry analytes and hematocrit at first handling. Generalized mixed models (fixed factors: group, time, recumbency; random factor: individual rhinoceros) were applied to compare longitudinal changes between free-ranging and game-farmed rhinoceroses immobilized with the higher etorphine dose (Groups 1 and 2), and between the two protocols tested in the game-farmed rhinoceroses (Groups 2 and 3). All animals were successfully immobilized, presented with normal lactate concentrations (<5 mmol/L), experienced no muscle tremors and recovered uneventfully. Hypoxemia and hypertension persisted throughout the immobilization in all groups. Acidemia and hypercarbia were absent in Group 1, but present in the game-farmed animals. The lower etorphine dose in Group 3 resulted in significantly longer induction times, however, tachycardia was not observed. SpO_2_ was higher for sternal vs. lateral recumbency. Semen-rich fractions were recovered following electro-stimulation in 46 out of the 62 animals. Our findings suggest that etorphine-medetomidine-midazolam provides effective immobilization with fewer side effects compared to previous reports in white rhinoceroses and is suitable for successful electro-ejaculation.

## Introduction

Similar to other rhinoceros species, southern white rhinoceros (*Ceratotherium simum simum*) numbers in the wild have shown a dramatic decrease associated with habitat loss and the ongoing poaching crisis (1). Biobanking of gametes and other genetic material and the use of assisted reproductive technologies have gained importance in combating the loss of genetic diversity of threatened or endangered species (2). Semen cryopreservation is an effective method to safeguard viable genetics. In wild, intractable species, electro-ejaculation is the most commonly utilized method of semen collection, but requires chemical restraint (3).

Potent, pure μ-receptor agonist opioids, such as etorphine hydrochloride, are routinely used for rhinoceros immobilization. However, these drugs are associated with dose-dependent hypoxemia, hypercarbia, acidemia, muscle rigidity, excitability, tachycardia, and systemic hypertension (4–12). These side effects are believed to be more pronounced when capturing free-ranging vs. captive animals due to a greater stress-induced catecholamine release and hyperlactatemia associated with the capture method (8, 13).

Partial antagonists such as butorphanol and diprenorphine are usually administered to reduce the etorphine-induced side effects and sedatives and/or tranquilizers are combined with opioids to achieve better muscle relaxation (6, 13). The butyrophenone tranquilizer azaperone is most frequently combined with etorphine (14) as it reduces opioid-induced hypertension (4, 10). Nevertheless, muscle rigidity, tachycardia, hypoxemia, hypercarbia, and acidemia still occur (6, 11, 14–16). Alpha_1_-receptors antagonists like azaperone, have detrimental dose-dependent effects on semen emission and were found to result in ejaculates with no or low sperm concentrations (17–19). Hence, alternative immobilization protocols that allow for safe and effective semen collection via electro-ejaculation (EE) in rhinoceroses are required. Free-ranging black rhinoceroses undergoing EE were successfully immobilized with a combination of etorphine-medetomidine-midazolam (20). Specific α_2_-adrenoreceptor agonists, including medetomidine and detomidine, provide dose-dependent sedation, analgesia, muscle relaxation and anxiolysis (21) and have a sympatholytic effect, which may reduce stress- and etorphine-induced catecholamine release and associated side effects (21). Additionally, as the ejaculatory reflex and semen emission are α-adrenergically mediated (22), α_2_-agonists were found to increase semen emission in felids (23) and stallions (24), and were suggested for the use in rhinoceroses undergoing electro-ejaculation (25). Midazolam, a benzodiazepine receptor agonist and potent sedative and muscle relaxant, was more recently incorporated in white rhinoceros immobilization protocols (12, 15, 26, 27). By reducing muscle spasms and rigidity (28), ventilation is improved and the risk of hyperthermia and capture myopathy reduced (29).

The goal of this study was to test the safety and efficacy of two different doses of an etorphine-medetomidine-midazolam combination for the immobilization of free-ranging and game-farmed white rhinoceroses undergoing semen collection using electro-stimulation.

To assess this combination, cardiovascular, respiratory, and acid-base statuses were monitored from induction through to reversal of the drugs. The primary aims were to investigate if: 1) free-ranging rhinoceroses experience greater cardiopulmonary side-effects compared to game-farmed rhinoceroses, 2) a higher etorphine dose results in greater cardiopulmonary side effects compared to a lower dose in combination with a higher medetomidine dose and 3) semen can be successfully collected from white rhinoceros bulls immobilized with etorphine-medetomidine-midazolam combinations by electro-ejaculation. Additionally, the effect of recumbency (lateral vs. sternal) was evaluated.

## Materials and Methods

### Animals and Study Areas

A total of 62 sexually mature male white rhinoceroses (*Cerothoterium simum simum*), whereof 14 free-ranging and 48 game-farmed, were immobilized for management purposes such as dehorning and breeding soundness evaluations. Immobilizations were carried out during the winter months of the southern hemisphere (June–August) in the provinces of Limpopo and the Northern Cape of South Africa. Animals were declared healthy based on behavior, body condition, and clinical examination following immobilization. Body condition was scored according to criteria based on subjective evaluation of fat deposits and muscle mass (good, fair, poor, very poor) as described previously (30). Ambient temperatures, recorded using the smart phone's weather widget for the respective location, typically ranged between 18–25°C in the Northern Cape and 25–32°C in Limpopo.

Animals in the Northern Cape were housed in closely supervised camps on a private game reserve with supplementary feeding consisting of hay, Lucerne, molasses and maize meal. For security reasons, the animals were housed in smaller enclosures during the night, whereas during the day, they had access to larger camps.

### Capture Method, Doses and Administration of Drugs

Rhinoceroses, location, immobilization protocols and recumbency position are summarized in Table 1. All doses were calculated based on standardized estimated weights (~2,000 kg). Fourteen free-ranging rhinoceroses (Group 1) in Limpopo were located using a fixed-wing aircraft. Once located, the animals were darted with a combination of ≈2.5 μg/kg etorphine (etorphine hydrochloride 9.8 mg/mL, Captivon; Wildlife Pharmaceuticals, Karino, South Africa), ≈2.5 μg/kg medetomidine (20 mg/mL, Kyron Laboratories, Benrose, South Africa), ≈25 μg/kg midazolam (midazolam hydrochloride 50 mg/mL, Dazonil; Wildlife Pharmaceuticals) and 1,500–1,700 IU hyaluronidase (lyophilised hyalase, Kyron Laboratories) delivered by dart gun (Pneu-Dart 389, Pneu-dart, Inc., Williamsport, Pennsylvania, USA) from a helicopter into the gluteal muscle-mass using 2.0 mL Pneu-dart darts (Pneu-dart Type C, Pneu-dart, Inc.) with 2.5-inch uncollared needles. The game-farmed animals (Groups 2 and 3) were darted on foot using the same dart gun and 3.0 mL Pneu-dart darts (Pneu-dart Type C, Pneu-dart, Inc.), either into the hindquarter or nuchal hump. Twenty-eight of these rhinoceroses (Group 2), were immobilized using the above-described combination of etorphine (M99; Novartis, Kempton Park, South Africa), medetomidine and midazolam and 20 rhinoceroses (Group 3) with ≈1 μg/kg etorphine, ≈5 μg/kg medetomidine, ≈25 μg/kg midazolam and 1,700 IU hyaluronidase.

**Table 1:** Immobilization protocols of free-ranging (Limpopo Province) and game-farmed (Northern Cape) rhinoceroses, showing capture method, drug doses, and routes of administration (i.m., intramuscularly; i.v., intravenously) in Groups 1, 2, and 3 with *n* = number of animals.

	**Group**		
	**1**	**2**	**3**
*n*	14	28	20
Origin management	Limpopo, free-ranging	Northern Cape, game-farmed	Northern Cape, game-farmed
Capture method	Darted from helicopter	Darted on foot	Darted on foot
Induction			
Etorphine i.m.	≈2.5 μg/kg	≈2.5 μg/kg	≈1 μg/kg
Medetomidine i.m.	≈2.5 μg/kg	≈2.5 μg/kg	≈5 μg/kg
Midazolam i.m.	≈25 μg/kg	≈25 μg/kg	≈25 μg/kg
Hyalase i.m.	1,500 IU	1,500 IU	1,700 IU
Butorphanol i.v. (auricular vein)^a^	≈2.5 μg/kg	≈2.5 μg/kg	-
Reversal			
Naltrexone i.v.	≈25 μg/kg	≈25 μg/kg	≈25 μg/kg
Atipamezole i.v. or i.v. and i.m.^b^	≈5–10 μg/kg	≈5–10 μg/kg	≈5–10 μg/kg
Recumbency position	Sternal	Sternal (*n* = 16), lateral (*n* = 9), sternal + lateral (*n* = 3)	Sternal (*n* = 12), lateral (*n* = 8)
Body condition^c^	Fair	Good	Good

a *Administered once recumbent*.

b *1/3 of the dose i.v. and 2/3 i.m*.

c *According to Keep (1971) (30)*.

Once immobilized, all animals were carefully approached, blindfolded and earplugs were applied. At first handling (t0), animals in Group 1 and 2 (higher dose of etorphine) received ≈2.5 μg/kg of butorphanol (50 mg/ml; Kyron Laboratories) intravenously in an auricular vein. Animals in Group 3 did not receive butorphanol as the etorphine-induced respiratory depression was expected to be less severe.

Time from darting to recumbency (induction time) and total time in recumbency were recorded. The time of reversal (t_R_) depended on the duration of the procedures.

Etorphine was antagonized with ≈25 μg/kg naltrexone HCL (Trexonil 50 mg/ml, Wildlife Pharmaceuticals) administered intravenously. To antagonize the medetomidine, 5–10 μg/kg atipamezole HCL (5 mg/mL mg; Atipamezole, Vtech, Midrand, South Africa or Antisedan, Zoetis, Sandton, South Africa, depending on availability) were administered intravenously (1/3 of the dose) and intramuscularly (2/3 of the dose; Table 1), respectively. Midazolam was not antagonized.

### Cardio-Respiratory Monitoring

Animals were continuously monitored for visible muscle tremors and vital signs were recorded at 5-min intervals from 5 min after first handling (t5) until reversal of immobilization (t_R_). Respiratory rate (f_R_) was determined by counting exhalations from the nostrils. A pulse-oximeter (SunTech Vet30E, Morrisville, NC, USA) using a Y-lingual sensor (AccuVet) was applied on a scarified edge of the pinna to record heart rate (HR), peripheral hemoglobin oxygen saturation (SpO_2_) and body temperature.

Oscillometric mean arterial (MAP), systolic (SAP), and diastolic (DAP) blood pressures were measured on the coccygeal artery at the base of the tail with a portable blood pressure monitoring device (SunTech, Vet30E, Morrisville, NC, USA), with an algorithm for indirect non-invasive measurement of blood pressure in horses. A 17–25 cm cuff was fitted around the tail (25 cm below the tail base and ~30–35 cm above the height of the right atrium) and single readings were recorded at t0 and t_R_. Serial measurements were not possible during the procedures due to the position of the rectal electro-ejaculator probe. It was estimated that the cuff was on heart level in animals in lateral position. In rhinoceroses in sternal position, however, indirect blood pressures were corrected to heart level using the formula Corrected Blood Pressure = ([distance in cm from center of cuff on tail to heart base/1.36] + actual coccygeal blood pressure) as previously described (31).

### Blood Sampling and Analyses

Arterial blood samples, collected from the auricular artery at t0, after butorphanol administration (t_B_; only Group 1 and 2) and at t_R_ in 1 mL heparinized syringes (Arterial Blood Sampler Aspirator, Radiometer Medical ApS, Denmark), were promptly analyzed using a portable blood gas analyzer (i-STAT Portable Clinical Analyzer and i-STAT CG4+ cartridges, Zoetis, Germany). Blood pH, partial pressure of oxygen (PaO_2_), partial pressure of carbon dioxide (PaCO_2_) and lactate concentration (Lac) were measured, while base excess (BE), bicarbonate (HCO${}_{3}^{-}$) and hemoglobin oxygen saturation (SaO_2_) were calculated by the analyzer based on an internal algorithm. All blood gas results were interpreted at a fixed body temperature of 37°C (alpha-stat analysis) by the analyzer. Additionally, sodium (Na), potassium (K), chloride (Cl), ionized calcium (iCa), glucose (Glu), urea (BUN), creatinine (Crea), and hematocrit (Hct) were measured at t0 (i-STAT Chem8+ cartridges, Zoetis, Germany).

### Electro-Ejaculation

All animals underwent electro-ejaculation (EE) by methods previously described elsewhere (32). The EE was performed with a portable, battery-powered electro-ejaculator (El Toro 3, Electronic Research Group, South Africa) and a specifically designed, custom-made rectal probe which the authors previously also applied in wild black rhinoceroses (20). Sets of stimuli, each consisting of 8–10 stimulations lasting 3 s, with a 2-s pause in between, were applied. With each set, the voltage was slowly increased to reach a maximum of 10 V and 277 mA.

### Data Analyses

Statistical analysis was performed with the software R version 3.6.1 (33). Data were assessed for normality by calculating descriptive statistics and plotting of histograms. Median and range were calculated for each parameter per group and interval plots were generated for descriptive purposes. Generalized mixed models (fixed factors: group, time, and recumbency; random factor: individual rhinoceros) were used to compare differences in cardiopulmonary and blood gas variables between free-ranging and game-farmed rhinoceroses (Groups 1 and 2), and between the two protocols tested in the game-farmed rhinoceroses (Groups 2 and 3). Due to the small sample size, non-parametric analyses were applied to compare induction time, time in recumbency, and blood chemistry results between the groups by using one-way ANOVA analysis on ranks. HR before the onset and 5 min after commencement of EE were compared using a Wilcoxon rank sum test. Differences were considered significant when *p* ≤ 0.05.

## Results

All animals were successfully immobilized, no emergency or corrective interventions were required, and no mortalities occurred. One animal in Group 1 and three animals in Group 3 failed to respond to the initial dart, which was due to malfunction of the dart. After re-administration using the same protocol, the animals responded similarly to animals in the same group. These individuals were excluded, however, from the induction time data.

Mean induction time of Group 3 (20.4 ± 4.5 min) was significantly longer (*p* = 0.001) than the times of Groups 1 (10.5 ± 2.2 min) and 2 (13.7 ± 4.5 min). Times in recumbency did not differ significantly between groups and were 38.6 ± 11.5 min, 39.3 ± 10.0 min, and 41.6 ± 13.7 min for Groups 1, 2 and 3, respectively. All 14 animals in Group 1, 16 in Group 2 and 12 in Group 3 were positioned in sternal, and nine in Group 2 and eight in Group 3 in lateral recumbency. Three animals in Group 2 were moved from sternal into lateral recumbency during the course of the anesthesia. Body position had a significant effect on SpO_2_ and non-invasive indirect blood pressures. SpO_2_, MAP, SAP, and DAP were higher in rhinoceroses positioned in sternal compared to lateral recumbency (*p* < 0.05; Supplementary Tables S5, S6).

After reversal, all animals recovered from the effects of the immobilizing drugs within 2.4 min (2.4 ± 1.1 min). Electro-stimulations started after 27.5 ± 11.7 min. Semen-rich fractions were recovered 15.48 ± 9.13 min after start of stimulation in 46 of the 62 animals. HR immediately before the onset of stimulations (52 [26–125] bpm) was lower (*p* < 0.001) than after commencement of stimulation (85.5 [30–142] bpm). Body temperature did not differ significantly between Groups at t0 (Group 1: 37.4°C [36.0–39.4], Group 2: 36.3°C [35.0–37.9]; Group 3: 36.4°C [36.3–37.2]), however, at tR body temperature was higher in Group 1 (38.0°C [36.0–39.7]) than in Group 2 (35.9°C [34.6–38.3], *p* = 0.004) and 3 (35.8°C [35.6–36.6], *p* = 0.004).

Descriptive analyses of HR, f_R_ and SpO_2_, MAP, SAP and DAP, pH, PaCO_2_, PaO2, HCO_3_-, BE, SaO_2_ and Lac, and Na, K, Cl, BUN, Crea, Glu, iCA, and Hct and statistical significances are shown in the Supplementary Tables S1–S6.

### Effect of Free-Ranging vs. Game-Farmed Rhinoceroses on Variables

**Figure 1:**
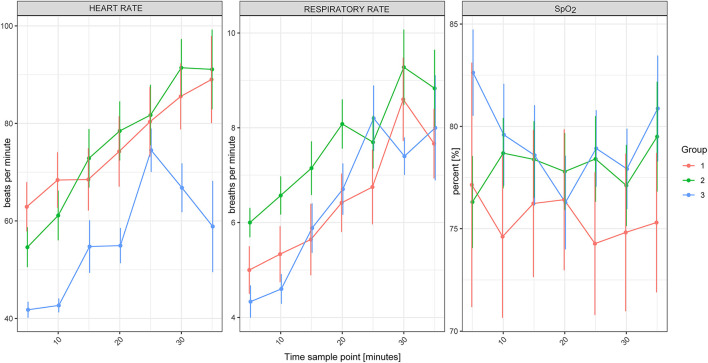
Blood pH, PaCO_2_ and HCO_3−_ of white rhinoceroses (*Ceratotherium simum simum*) per group: Group 1, free-ranging (*n* = 14), immobilized with ≈2.5 μg/kg etorphine (high dose), ≈2.5 μg/kg medetomidine, ≈25 μg/kg midazolam and 1,500–1,700 IU hyaluronidase; Group 2, game-farmed (*n* = 28), immobilized with ≈2.5 μg/kg etorphine (high dose), ≈2.5 μg/kg medetomidine, ≈25 μg/kg midazolam and 1,500–1,700 IU hyaluronidase and Group 3, game-farmed animals (*n* = 20) ≈1 μg/kg etorphine (low dose), ≈5 μg/kg medetomidine and ≈25 μg/kg midazolam. Time sampling points: t0) at first handling, t_B_) after butorphanol administration and t_R_) at reversal. Data presented as Median (range).

**Figure 2:**
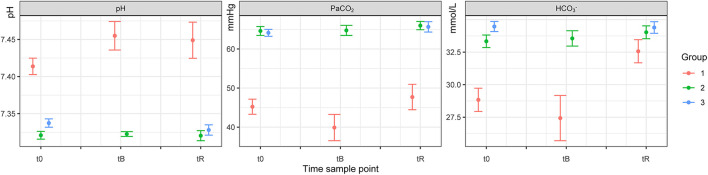
Heart rate, respiratory rate and SpO_2_ of white rhinoceroses (*Ceratotherium simum simum*) per group: Group 1, free-ranging (*n* = 14), immobilized with ≈2.5 μg/kg etorphine (high dose), ≈2.5 μg/kg medetomidine, ≈25 μg/kg midazolam and 1,500–1,700 IU hyaluronidase; Group 2, game-farmed (*n* = 28), immobilized with ≈2.5 μg/kg etorphine (high dose), ≈2.5 μg/kg medetomidine, ≈25 μg/kg midazolam and 1,500–1,700 IU hyaluronidase and Group 3, game-farmed animals (*n* = 20) ≈1 μg/kg etorphine (low dose), ≈5 μg/kg medetomidine and ≈25 μg/kg midazolam. Time sampling points: five to 35 min after recumbency in 5-min intervals. Data presented as Median (range).

There was no significant effect of Group on HR, f_R_ and SpO_2_ between free-ranging and game-farmed rhinoceroses immobilized with the same protocol (Figure 1). Oscillometric MAP was higher in Group 2 compared to Group 1 (*p* = 0.039). Group had a significant effect on arterial blood gases. The pH was higher in Group 1 compared to 2 (*p* < 0.001) and PaCO_2_, HCO_3_-, and BE were lower in Group 1 compared to Group 2 (*p* < 0.001 all variables, except for BE *p* = 0.011; Figure 2). K, Cl and Crea concentrations were higher in Group 1 compared to Group 2 (*p* < 0.001) and iCa concentrations were lower in Group 1 than Group 2 (*p* < 0.001).

Time had a significant effect on HR and f_R_, with both increasing over time (*p* < 0.001).

### Effect of Etorphine and Medetomidine Doses in Game-Farmed Rhinoceroses

Model based analysis revealed that there was a significant effect of Group on HR and f_R_, which were higher in Group 2 (high etorphine, low medetomidine) compared to Group 3 (low etorphine, high medetomidine). There was no significant effect of Group on oscillometric blood pressure measurements and arterial blood gas concentrations. Na, Cl and iCa concentrations were lower in Group 3 than Group 2 (*p* = 0.012, 0.005, and 0.042, respectively). Glu concentrations were higher in Group 3 compared to Group 2 (*p* = 0.004), whereas Hct was higher in Group 2 than Group 3 (*p* < 0.001).

Again, HR and f_R_ increased over time (*p* < 0.001). Lac concentrations of Group 3 increased over time (*p* = 0.002).

## Discussion

Immobilizations with both combinations of etorphine-medetomidine-midazolam were uncomplicated and without visible muscle tremors, but significant changes in cardiorespiratory and blood gas variables occurred. Median respiratory rates, indirect oscillometric blood pressure and blood oxygenation in all three groups and during each sampling time point deviated from those recorded in unrestrained standing captive white rhinoceroses (31). This finding is consistent with other studies where white rhinoceroses were immobilized with etorphine-combinations (6, 11, 12, 14, 16).

Hypoxemia, hypercarbia, and respiratory and metabolic (lactic acid) acidemia are consistent findings in white rhinoceroses immobilized with etorphine-based combinations (6, 11, 12, 14–16, 26), and caused by several factors including pulmonary hypertension, hypoventilation, and hypermetabolism associated with the μ-receptor-related and sympathetic effects of the etorphine (34–36).

Hypoxemia was present in all groups and similar to previous reports in etorphine-immobilized white rhinoceroses (6, 7, 27). However, hypercarbia and respiratory acidemia were only present in the game-farmed rhinoceroses (Groups 2 and 3) and similar, or less profound, compared to studies where etorphine was combined with either azaperone (6, 11, 14, 16) or midazolam (26, 27). Hypoventilation is common and results from ventilation–perfusion mismatch, a central inhibitory effect of etorphine on the respiration, respiratory muscle rigidity, increased upper airway resistance and the reduced expansion of the lungs due to the pressure of the viscera on the diaphragm (6, 11, 12, 15, 27, 37, 38). Positioning is an important factor in this regard. Similar to other studies, oxygenation was improved in rhinoceroses positioned in sternal compared to lateral recumbency indicating a decreased dead-space ventilation (12, 13, 39). Interestingly, in the free-ranging rhinoceroses (Group 1), PaCO_2_ and pH were within the reference ranges of unsedated animals [44.4–53.7 mmHg and 7.346–7.431 (31)]. The lower body condition and better fitness of the free-ranging compared to the game-farmed rhinoceroses, may have improved ventilation (38). The generally lower PaCO_2_ and higher pH in the rhinoceroses of all groups compared to previous reports in etorphine-azaperone immobilized white rhinoceroses (6, 7, 11, 14–16, 27, 40) indicate that ventilation was better, despite an apparently lower f_R._ Similar results were previously obtained in black rhinoceroses immobilized with etorphine-medetomidine-midazolam (20). The absence of muscle tremors and an improved muscle relaxation induced by medetomidine and midazolam possibly resulted in a decreased chest wall rigidity, reduced global ventilation-to-perfusion mismatch and improved gas exchange through enhanced respiratory excursions (14). The improved muscle relaxation in this study, may additionally explain the consistent gradual increase of the f_R_ over time, compared to etorphine-azaperone immobilized rhinoceroses where f_R_ increased only transiently after the administration of butorphanol (41). In support of these assumptions, deeper breaths in animals immobilized with etorphine-medetomidine-midazolam compared to etorphine-azaperone were observed by one of the authors (BT), who has extensive experience in white rhinoceros immobilization. However, further studies comparing respiratory flows and volumes of rhinoceroses immobilized with etorphine-medetomidine-midazolam and etorphine-azaperone are required to confirm this subjective perception. Even though rhinoceroses of Group 3 did not receive butorphanol, their hypoxemia was not worse compared to the two other groups; a result that was likely due to a lower etorphine dose. Interestingly, in this group, lactate concentrations increased over time. Possibly, butorphanol alleviated this increase and the associated hypoxemia in animals of Groups 1 and 2 (6) and needs to be tested. However, lactic acidosis was absent in the rhinoceroses of all three groups as the concentrations remained within normal limits for the species (42), which we additionally observed in black rhinoceroses previously (20). These results may reflect the rapid and smooth induction achieved with the drug combination used (6), even in rhinoceroses darted from the helicopter.

Severe tachycardia and systemic hypertension are consistent findings in white rhinoceroses immobilized with etorphine combinations (8) and are believed to be a result of an etorphine-induced upregulated sympathetic activity (7).

The HR was elevated in rhinoceroses of Groups 1 and 2, which is in agreement with previous findings (7, 15). However, in both groups, the HR was initially lower than reported with etorphine-azaperone [130–144 bpm (15)] and in Group 3 (lower doses of etorphine), the HR was largely within normal ranges quoted for unsedated rhinoceroses (32–42 bpm) (31). Medetomidine is known to cause bradycardia in response to vasoconstriction (reflex-bradycardia), through a reduced sympathetic tone and suppression of the cardiovascular center (43, 44). Thus, it may have compensated for the effects of the etorphine (31). However, the lower etorphine dose likely caused less etorphine-induced sympathetic stimulation and associated tachycardia. Further research is required to differentiate the effects of the higher medetomidine dose from the lower etorphine dose administered in rhinoceroses of Group 3. Nevertheless, the results demonstrate a desirable effect of this protocol on HR with longer induction times as a disadvantage. The protocol therefore appears suited for rhinoceroses under captive or controlled settings rather than free-ranging individuals.

In contrast to previous studies in etorphine-azaperone immobilized white rhinoceroses (10, 15), HR gradually increased over time in all three groups. However, they remained similar or lower compared to previously reported values (11, 15). This gradual increase in HR may reflect a decrease in systemic medetomidine concentrations, which is known to occur 10–20 min after intramuscular administration (45). Additionally, sympathetic activation caused by the EE, may have contributed since the HR increased after the commencement of stimulations. Besides the direct cardiovascular- and sympathetic-tone reducing effects of medetomidine, its sedative properties may also have influenced HR by providing a deeper anesthesia. Further research in a more controlled setting is needed to elucidate the proposed effects of the medetomidine, as well as tease out the effects of the other drugs and procedures in the rhinoceros.

In this study, indirect blood pressures varied greatly amongst the individuals and clinical hypertension was observed in less than half of the rhinoceroses [reference range for unsedated white rhinoceroses: MAP: 92–130 mmHg; SAP: 123–157 mmHg; DAP: 66–89 mmHg (30)]. Medetomidine, which causes initial peripheral vasoconstriction (21, 43), may have influenced readings and possibly contributed to these differences. The higher MAP in Group 2 compared to Group 1 was surprising. Possibly the confinement to smaller enclosures prior to the immobilization may have increased stress status of the game-farmed rhinoceroses (46). Interestingly, indirect blood pressure measurements were higher in animals in sternal recumbency. Vascular compression by the viscera in lateral recumbency may reduce venous return and thus, blood pressure (47). Unfortunately, serial measurement of indirect blood pressure on the tail during EE was not possible, given the position of the rectal probe. Continuous measurement of direct arterial blood pressure would be advisable to further investigate the cardiovascular effects associated with this immobilization protocol in future studies.

Rhinoceroses from Group 3 had a lower Hct compared to Group 2. An increased Hct is proposed to be associated with etorphine-induced catecholamine release (48). On the other hand, the higher glucose concentration in rhinoceroses of Group 3, is possibly related to a higher medetomidine dose, which has been shown to increase blood glucose concentrations in domestic animals (49). Differences in blood electrolyte and Crea concentrations likely reflected differences in hydration and nutritional status of the animals (50). Higher iCa concentrations in the game-farmed than in the free-ranging animals conforms with the findings of a previous report (51). However, significant differences in Na, Cl and iCa between Group 2 and 3 (game-farmed animals under same environmental conditions) may be related to the immobilization protocol.

The inclusion of α2-adrenoreceptor agonists in immobilization protocols of white rhinoceroses undergoing electro-ejaculation, has previously been suggested (25). In this study, viable semen samples were collected from 74% of the bulls, confirming the suitability of these immobilization protocols for EE.

### Study Limitations

Data collection time points were pre-determined by the planned procedures. Rhinoceroses of Group 3 received no butorphanol whereas those in Groups 1 and 2 (high etorphine) did. Butorphanol is commonly administered to reduce the etorphine-induced side effects, which were expected to be more prominent in Groups 1 and 2. Moreover, the dose of etorphine was lowered in Group 3 while the medetomidine dose was increased to achieve sufficient immobilization, making it difficult to distinguish the effects of the two individual drugs. Within Groups 2 and 3, animals were either placed in lateral or sternal position, while all animals in Group 1 were positioned sternally. We dealt with this issue by including the main confounder, recumbency, as fixed effects in the generalized mixed models together with the effect of time.

Different environmental conditions at the two locations as well as the differences in body condition and nutrition may also have influenced our findings.

Additionally, non-invasive blood pressure devices have not been validated for the use in rhinoceroses and positioning of the cuff proved rather challenging due to the anatomy of the tail. Instead, the equine-specific algorithm (VetBP; SunTech Medical Inc., NC, USA) were applied. However, no published information was found on the reliability and accuracy of non-invasive blood pressure monitored with this equipment in horses. The blood pressures were furthermore corrected to heart level in animals in sternal position using an estimated distance between cuff and heart base and paired measurements were still able to register changes in blood pressure during the immobilization with etorphine-medetomidine-midazolam. Differences between groups were only seen in MAP, which is the most reliable non-invasive method of measuring blood pressure in horses (52). Scarified SpO_2_ readings have not been validated in rhinoceroses. However, SpO_2_ readings were consistent with SaO_2._

## Conclusions

Immobilizations with etorphine-medetomidine-midazolam were uncomplicated, resulted in mostly acceptable blood gas and cardiorespiratory parameters and allowed for successful semen collection via electro-ejaculation. Severe hypoxemia as previously described indicates a need for oxygen supplementation during semen collection. These results suggest that etorphine in combination with medetomidine and midazolam, provides a safe and effective immobilization protocol for free-ranging white rhinoceroses and may be recommended for semen collection using electro-ejaculation.

## Data Availability Statement

The original contributions presented in the study are included in the article/Supplementary Material, further inquiries can be directed to the corresponding authors.

## Ethics Statement

The animal study was reviewed and approved by the University of Pretoria Animal Ethics and Research Committee.

## Author Contributions

IL, JM, and IL-B designed the experiment together with BT. BT and CB took veterinary care of the rhinoceroses and BT and ST collected the arterial blood samples. Data was collected and prepared by JM, and analyzed by FP and JM. JM prepared the manuscript together with HB, FP, and IL. All authors approved the final manuscript.

## Funding

This research project was entirely funded by Rhino Force SA NPC.

## Conflict of Interest

The authors declare that the research was conducted in the absence of any commercial or financial relationships that could be construed as a potential conflict of interest.

## Publisher's Note

All claims expressed in this article are solely those of the authors and do not necessarily represent those of their affiliated organizations, or those of the publisher, the editors and the reviewers. Any product that may be evaluated in this article, or claim that may be made by its manufacturer, is not guaranteed or endorsed by the publisher.

## References

[1] Emslie R. Ceratotherium simum. IUCN Red List Threat Species. (2020). e.T4185A45.

[2] Comizzoli P. Banking efforts and new advances in male fertility preservation for rare and endangered species. Asian J Androl. (2015) 17:640–5. 10.4103/1008-682X.153849 25966625 PMC4492057

[3] Prieto MT Hildebrandt TB Saragusty J. Sperm cryopreservation in wild animals. Eur J Wildl Res. (2014) 60:851–64. 10.1007/s10344-014-0858-4

[4] Hattingh J Knox CM Raath JP. Arterial blood-pressure and blood-gas composition of white rhinoceroses under etorphine anesthesia. S Afr J Wildl Res. (1994) 24:12–4.

[5] Bush M Citino SSB Grobler D. Improving Cardio-Pulmonary Function for a Safer Anesthesia of White Rhinoceros (Ceratotherium Simum): Use of Opiate Cocktails to Influence Receptor Effects. Proc AAZV, AAWV, AZA/NAG Jt Conf [Internet]. (2005) 259–60. Available online at: https://scholar.google.com/scholar?start=160&q=Citino&hl=en&as_sdt=0:10#4

[6] Buss P Olea-Popelka F Meyer L Hofmeyr J Mathebula N Kruger M. Evaluation of cardiorespiratory, blood gas, and lactate values during extended immobilization of white rhinoceros (*Ceratotherium simum*). J Zoo Wildl Med. (2015) 46:224–33. 10.1638/2014-0089R.1 26056872

[7] Boesch JM Gleed RD Buss P Hofmeyr M Tordiffe A Zeiler G. Effects of a supplemental etorphine dose on pulmonary artery pressure and cardiac output in immobilized, boma-habituated white rhinoceros (*Cerathoterium simum*): a preliminary study. J Zoo Wildl Med. (2018) 49:849–55. 10.1638/2017-0120.1 30592907

[8] De Lange S Fuller A Haw A Hofmeyr M Buss P Miller M. Tremors in white rhinoceroses (*Ceratotherium simum*) during etorphine–azaperone immobilisation. J S Afr Vet Assoc. (2017) 88:1466. 10.4102/jsava.v88i0.1466 28281770 PMC6138155

[9] Kock MD Morkel P Atkinson M Foggin C. Chemical immobilization of free-ranging white rhinoceros (*Ceratotherium simum simum*) in Hwange and Matobo National Parks, Zimbabwe, using combinations of etorphine (M99), fentanyl, xylazine, and detomidine. J Zoo Wildl Med. (1995) 26:207–19.

[10] Meyer LCR Fuller A Hofmeyr M Buss P Miller M Haw A. Use of butorphanol and diprenorphine to counter respiratory impairment in the immobilised white rhinoceros (*Ceratotherium simum*). J S Afr Vet Assoc. (2018) 89:e1–e8. 10.4102/jsava.v89i0.1683 30456980 PMC6244275

[11] Miller M Buss P Joubert J Mathebula N Kruger M Martin L. Use of butorphanol during immobilization of free-ranging white rhinoceros (*ceratotherium simum*). J Zoo Wildl Med. (2013) 44:55–61. 10.1638/1042-7260-44.1.55 23505703

[12] Wenger S Boardman W Buss P Govender D Foggin C. The cardiopulmonary effects of etorphine, azaperone, detomidine, and butorphanol in field-anesthetized white rhinoceroses (*Ceratotherium simum*). J Zoo Wildl Med. (2007) 38:380–7. 10.1638/2006-0038R.1 17939346

[13] Morkel PV Radcliffe RW Jago M du Preez P Flaminio MJ Nydam DV. Acid-base balance and ventilation during sternal and lateral recumbency in field immobilized black rhinoceros (*Diceros bicornis*) receiving oxygen insufflation: a preliminary report. J Wildl Dis. (2010) 46:236–45. 10.7589/0090-3558-46.1.236 20090037

[14] Portas T. A review of drugs and techniques used for sedation and anaesthesia in captive rhinoceros species. Aust Vet J. (2004) 82:542–9. 10.1111/j.1751-0813.2004.tb11196.x 15478725

[15] Buss P Miller M Fuller A Haw A Wanty R Olea-Popelka F. Cardiovascular effects of etorphine, azaperone, and butorphanol combinations in chemically immobilized captive white rhinoceros (*Ceratotherium simum*). J Zoo Wildl Med. (2016) 47:834–43. 10.1638/2015-0298.1 27691950

[16] Boardman WSJ Caraguel CGB Raath JP Van Zijll Langhout M. Intravenous butorphanol improves cardiopulmonary parameters in game-ranched white rhinoceroses (*Ceratotherium simum*) immobilized with etorphine and azaperone. J Wildl Dis. (2014) 50:849–57. 10.7589/2013-12-327 25105814

[17] Meltzer DGA Van Vuuren M Bornman M. The suppression of electro-ejaculation in the Chacma baboon (*Papio ursinus*) by azaperone. J S Afr Vet Assoc. (1988) 59:53. 3361563

[18] Kimsakulvech S Suttiyotin P Pinyopummin A. Effects of alpha1-adrenoceptor antagonist (*Tamsulosin*) on incident of ejaculation and semen quality in the goat. Andrologia. (2015) 47:354–9. 10.1111/and.12270 24684217

[19] Serdar G Yusuf K Selim K Hasret T Hidayet C Bedrettin S. Recovery of abnormal ejaculation by intermittent tamsulosin treatment. J Urol. (2006) 175:650–2; discussion 652–3. 10.1016/S0022-5347(05)00157-6 16407016

[20] Meuffels J Lueders I Bertschinger HJ Luther-Binoir I Pohlin F Gerber L. Cardiopulmonary parameters and arterial blood gases during etorphine-medetomidine-midazolam immobilization in free-ranging black rhinoceroses (*Diceros bicornis*) undergoing electro-ejaculation – a preliminary study. Front Vet Sci. (2021) 8:740614. 10.3389/fvets.2021.740614 34926635 PMC8674947

[21] Sinclair M. A review of the physiological effects of alpha2-agonists related to the clinical use of medetomidine in small animal practice. Can Vet J. (2003) 44:885–97. 14664351 PMC385445

[22] Giuliano F Clement P. Neuroanatomy and physiology of ejaculation. Annu Rev Sex Res. (2005) 16:190–16. 16913292

[23] Zambelli D Cunto M Prati F Merlo P. Effects of ketamine or medetomidine administration on quality of electroejaculated sperm and on sperm flow in the domestic cat. Theriogenology. (2007) 68:796–803. 10.1016/j.theriogenology.2007.06.008 17662381

[24] Cavalero TMS Papa FO Schmith RA Scheeren VFC Canuto LEF Gobato MLM. Protocols using detomidine and oxytocin induce ex copula ejaculation in stallions. Theriogenology. (2019) 140:93–8. 10.1016/j.theriogenology.2019.08.024 31454723

[25] Walzer C Pucher H Schwarzenberger F. A restraint chute for semen collection in white rhinoceros (*Ceratotherium simum simum*) –preliminary results. In: European Association of Zoo and Wildlife Veterinarians. Paris;. (2000) p. 7–10.

[26] Pohlin F Buss P Hooijberg EH Meyer LCR. Midazolam alters acid-base status less than azaperone during the capture and transport of southern white rhinoceroses (*Ceratotherium simum simum*). Animals. (2020) 10:1323. 10.3390/ani10081323 32751806 PMC7460343

[27] Van Zijll Langhout M Caraguel CGB Raath JP Boardman WSJ. Evaluation of etorphine and midazolam anesthesia, and the effect of intravenous butorphanol on cardiopulmonary parameters in game-ranched white rhinoceroses (*ceratotherium simum*). J Zoo Wildl Med. (2016) 47:827–33. 10.1638/2015-0167.1 27691936

[28] Plumb D. Midazolam. In: Plum D editor. Veterinary Drug Handbook. 2nd Edn. Ames, IA: Iowa University State Press (1995). p. 457–9.

[29] Radcliffe R Morkel P. Rhinoceroses. In: West G Heard D Caulkett N editors. Zoo animal and wildlife immobilization and anesthesia. 2nd ed. Ames (IA): Wiley Blackwell;. (2014) p. 741–771. 10.1002/9781118792919.ch54

[30] Keep M. Observable criteria for assessing the physical condition of the white rhinoceros *Ceratotherium simum* in the field. Lammergeyer. (1971) 13:15–28.

[31] Citino SB Bush M. Reference cardiopulmonary physiologic parameters for standing, unrestrained white rhinoceroses (*Ceratotherium simum*). J Zoo Wildl Med. (2007) 38:375–9. 10.1638/2006-0007R1.1 17939345

[32] Roth TL Stoops MA Atkinson MW Blumer ES Campbell MK Cameron KN. Semen collection in rhinoceroses (*Rhinoceros unicornis, Diceros bicornis, Ceratotherium simum*) by electroejaculation with a uniquely designed probe. J Zoo Wildl Med. (2005) 36:617–27. 10.1638/05-019.1 17312718

[33] R Development Core Team. R: A Language and Environment for Statistical Computing. Vienna, Austria: R Foundation for Statistical Computing (2019).

[34] Meyer RCR Hetem SS Mitchell D Fuller A. Hypoxia following etorphine administration in goats (*Capra hircus*) results more from pulmonary hypertension than from hypoventilation. BMC Vet Res. (2015) 11:18. 10.1186/s12917-015-0337-5 25644810 PMC4322799

[35] Buss P Miller M Fuller A Haw A Stout E Olea-Popelka F. Postinduction butorphanol administration alters oxygen consumption to improve blood gases in etorphine-immobilized white rhinoceros. Vet Anaesth Analg. (2018) 45:57–67. 10.1016/j.vaa.2017.03.008 29242121

[36] Radcliffe RW Jago M Morkel PV Morkel E du Preez P Beytell P. The pulmonary and metabolic effects of suspension by the feet compared with lateral recumbency in immobilized black rhinoceroses (*Diceros bicornis*) captured by aerial darting. J Wildl Dis. (2021) 57:357–67. 10.7589/2019-08-202 33822147

[37] Yaksh T Wallace M. Opioids, analgesia, and pain management. In: Brunton L Chabner B Knollman B editors. Goodman & Gilman's the Pharmacological Basics of Therapeutics, 12th Edn. New York, NY: McGraw Hill Medical (2011). p. 481–523.

[38] Pelosi P Croci M Ravagnan I Tredici S Pedoto A Lissoni A. The effects of body mass on lung volumes, respiratory mechanics, and gas exchange during general anesthesia. Anesth Analg. (1998) 87:654–60. 10.1213/00000539-199809000-00031 9728848

[39] Radcliffe RW Morkel PV Jago M Taft AA Du Preez P Miller MA. Pulmonary dead space in free-ranging immobilized black rhinoceros (*Diceros bicornis*) in Namibia. J Zoo Wildl Med. (2014) 45:263–71. 10.1638/1042-7260-45.2.263 25000686

[40] Miller M Buss P. Rhinoceridae (Rhinoceroses). In: Miller RE Fowler ME editors. Fowler's Zoo and Wild Animal Medicine: Current Therapy. 8th Edn. New York, NY: Elsevier (2015). p. 538–547. 10.1016/B978-1-4557-7397-8.00055-4

[41] Haw A Hofmeyr M Fuller A Buss P Miller M Fleming G. Butorphanol with oxygen insufflation corrects etorphine-induced hypoxaemia in chemically immobilized white rhinoceros (*Ceratotherium simum*). BMC Vet Res. (2014) 10:253. 10.1186/s12917-014-0253-0 25315767 PMC4205281

[42] Cole G Tordiffe A Steenkamp G. Assessment of a portable lactate meter for field use in the white rhinoceros (*Ceratotherium simum*). Onderstepoort J Vet Res. (2017) 84:e1–e10. 10.4102/ojvr.v84i1.1399 29227129 PMC8552300

[43] England GCW Clarke KW. Alpha2 adrenoceptor agonists in the horse—A review. Br Vet J. (1996) 152:641–57. 10.1016/S0007-1935(96)80118-7 8979422

[44] Knaus AE Muthig V Schickinger S Moura E Beetz N Gilsbach R. Alpha2-adrenoceptor subtypes – unexpected functions for receptors and ligands derived from gene-targeted mouse models. Neurochem Int. (2007) 51:277–81. 10.1016/j.neuint.2007.06.036 17664025

[45] Dugdale AHA Beaumont G Bradbrook C Gurney M. alpha2 adrenoreceptor agonists. In: Veterinary Anaesthesia: Principles to Practice. Second. Chichester, UK: John Wiley & Sons Ltd.;. (2020) p. 64–71.

[46] Vincent IC Michell AR Leahy RA. Non-invasive measurement of arterial blood pressure in dogs: a potential indicator for the identification of stress. Res Vet Sci. (1993) 54:195–201. 10.1016/0034-5288(93)90056-L 8460259

[47] Schatzmann U Hotz D Stauffer J Hess N. General anaesthesia in the horse in the upright position: a comparison with lateral recumbency using clinical and respiratory data. Vet Anaesth. (1991) 18:291–9. 10.1111/j.1467-2995.1991.tb00566.x

[48] Pohlin F Hooijberg EH Buss P Huber N Viljoen FP Blackhurst D. A comparison of hematological, immunological, and stress responses to capture and transport in wild white rhinoceros bulls (*Ceratotherium simum simum*) supplemented with azaperone or midazolam. Front Vet Sci. (2020) 7:569576. 10.3389/fvets.2020.569576 33195552 PMC7606872

[49] Ranheim B Horsberg NE Soli K Ryeng A Arnemo JM. The effects of medetomidine and its reversal with atipamezole on plasma glucose, cortisol and noradrenaline in cattle and sheep. J Vet Pharmacol Ther. (2000) 23:379–387. 10.1046/j.1365-2885.2000.00291.x 11168916

[50] Carlson GP. Fluid, Electrolyte, and Acid-Base Balance. In: Kaneko J. Harvey JW. Bruss M. editors. Clinical Biochemistry of Domestic Animals. 5th ed. Cambridge: Academic Press;. (1997) p. 485–516. 10.1016/B978-012396305-5/50019-1

[51] Trivedi S Burnham CM Capobianco CM Boshoff C Zheng Y Pettiglio JW. Analysis of blood biochemistry of free ranging and human-managed southern white rhinoceros (*Ceratotherium simum simum*) using the i-STAT Alinity v®. Vet Med Int. (2021) 2021:2665956. 10.1155/2021/2665956 34336179 PMC8315869

[52] Olsen E Pedersen TLS Robinson R Haubro Andersen P. Accuracy and precision of oscillometric blood pressure in standing conscious horses. J Vet Emerg Crit Care. (2016) 26:85–92. 10.1111/vec.12411 26488617

