# Supplementary material for: Arterial Blood Gases and Cardiorespiratory Parameters in Etorphine-Medetomidine-Midazolam Immobilized Free-Ranging and Game-Farmed Southern White Rhinoceroses (Ceratotherium simum simum) Undergoing Electro-Ejaculation
Source: Front Vet Sci. 2022 Apr 27;9:862100. doi: 10.3389/fvets.2022.862100 (PMC9094040; doi:10.3389/fvets.2022.862100)
Supplement: Supplementary file 1 [file Data_Sheet_1.docx]

# SUPPLEMENTARY MATERIAL

**Table S1:** Heart rate (HR), respiratory rate (f_R_) and peripheral hemoglobin oxygen saturation (SpO_2_) measured by pulse oximetry from the first measurement (t5) until maximum 55 min (t55) per group: Group 1, free-ranging (*n*=14), immobilized with ≈2.5 µg/kg etorphine (high dose), ≈2.5 µg/kg medetomidine, ≈25 µg/kg midazolam and 1500-1700 IU hyaluronidase; Group 2, game-farmed (*n*=28), immobilized with ≈2.5 µg/kg etorphine (high dose), ≈2.5 µg/kg medetomidine, ≈25 µg/kg midazolam and 1500-1700 IU hyaluronidase and Group 3, game-farmed animals (*n*=20) ≈1 µg/kg etorphine (low dose), ≈5 µg/kg medetomidine, ≈25 µg/kg midazolam and 1700 IU hyaluronidase. Data presented as Median (range) with *number of animals*.

| Variable (Unit) | Group | Time | | | | | | | | | | | |
| --- | --- | --- | --- | --- | --- | --- | --- | --- | --- | --- | --- | --- | --- |
|  |  | t5 | t10 | t15 | t20 | t25 | t30 | t35 | t40 | t45 | t50 | t55 |  |
| HR | 1 | 62  (37-100)  *10* | 64  (39-108)  *13* | 63  (35-112)  *14* | 71  (50-134)  *12* | 76  (51-127)  *12* | 89  (54-128)  *11* | 93  (48-138)  *10* | 54, 64  *2* | 59, 90  *2* | 46  *1* | 114  *1* |  |
|  | 2 | 49  (34-112)  *23* | 55  (33-135)  *27* | 64  (37-142)  *27* | 74  (35-147)  *26* | 82  (34-162)  *22* | 91  (55-145)  *22* | 89  (50-147)  *12* | 81  (59-123)  *11* | 86  (51-137)  *9* | 101  (68-119)  *3* | 102,112  *2* |  |
|  | 3 | 45  (32-52)  *15* | 42  (34-52)  *16* | 47  (35-93)  *7* | 52  (35-84)  *17* | 72  (54-110)  *16* | 66  (40-110)  *15* | 48  (37-108)  *8* | 39, 72  *4* | 47, 107  *2* | 70  *1* |  |  |
| f_R_ | 1 | 5 (3-8)  *13* | 5 (3-9)  *14* | 5 (3-12)  *14* | 7 (3-10)  *12* | 7 (4-11)  *12* | 9 (4-13)  *11* | 8 (3-11)  *10* | 4  *2* | 4, 5  *2* | 4  *1* | 10  *1* |  |
|  | 2 | 6 (3-8)  *23* | 7 (3-11)  *27* | 7 (3-15)  *27* | 8 (4-15)  *25* | 7 (4-13)  *23* | 9 (3-15)  *22* | 9 (3-13)  *12* | 9 (5-14)  *11* | 10 (6-13)  *9* | 8 (7-9)  *3* | 7,14  *2* |  |
|  | 3 | 4 (3-7)  *15* | 4 (3-6)  *17* | 6 (4-12)  *17* | 7 (3-12)  *17* | 8 (5-16)  *16* | 7 (5-11)  *15* | 8 (3-12)  *8* | 7 (5-9)  *4* | 6, 10  *2* | 8  *1* |  |  |
| SpO_2_ | 1 | 74  (57-98)  *7* | 72  (55-97)  *13* | 74  (52-97)  *13* | 81  (56-90)  *12* | 80  (52-88)  *11* | 72  (47-89)  *11* | 77  (50-87) *10* | 70, 83  *2* | 72, 88  *2* | 89  *1* | 80  *1* |  |
|  | 2 | 78  (54-90)  *23* | 80  (58-96)  *27* | 80  (60-95)  *26* | 78  (55-94)  *25* | 78  (53-95)  *22* | 78  (62-90)  *22* | 84  (67-89)  *12* | 81  (68-88)  *11* | 81  (70-87)  *9* | 88  (87-88)  *2* | 87  *1* |  |
|  | 3 | 86  (67-94)  *16* | 78  (56-98)  *17* | 78  (59-98)  *17* | 74  (59-93)  *18* | 77  (70-90)  *16* | 76  (68-89)  *15* | 83  (70-88)  *8* | 87  (75-93)  *4* | 64, 97  *2* | 96  *1* |  |  |

**Table S2:** The mean arterial blood pressure (MAP), systolic blood pressure (SAP) and diastolic blood pressure (DAP) first handling (t0) and immediately before reversal (t_R_) per group: Group 1, free-ranging (*n*=14), immobilized with ≈2.5 µg/kg etorphine (high dose), ≈2.5 µg/kg medetomidine, ≈25 µg/kg midazolam and 1500-1700 IU hyaluronidase; Group 2, game-farmed (*n*=28), immobilized with ≈2.5 µg/kg etorphine (high dose), ≈2.5 µg/kg medetomidine, ≈25 µg/kg midazolam and 1500-1700 IU hyaluronidase and Group 3, game-farmed animals (*n*=20) ≈1 µg/kg etorphine (low dose), ≈5 µg/kg medetomidine, ≈25 µg/kg midazolam and 1700 IU hyaluronidase. All blood pressures of animals in sternal recumbency were corrected with the formula Corrected Blood Pressure = ([distance in cm from center of cuff on tail to heart base/1.36] + actual coccygeal blood pressure). Data presented as Median (range) with *number of animals*.

| Variable (Unit) | Group | Time | | |
| --- | --- | --- | --- | --- |
|  |  | t0 | t_R_ | |
| MAP | 1 | 154 (81-196)  *11* | 138 (97-194)  *13* | |
|  | 2 | 166 (66-195)  *26* | 161 (81-222)  *26* | |
|  | 3 | 159 (65-205)  *19* |  | |
| SAP | 1 | 207 (101-257)  *11* | 204 (117-254)  *13* | |
|  | 2 | 200 (105-249)  *26* | 210 (145-277)  *26* | |
|  | 3 | 211 (92-255)  *19* |  | |
| DAP | 1 | 141 (75-178)  *11* | 130 (88-183)  *26* | |
|  | 2 | 154 (60-178)  *26* | 150 (55-207)  *19* | |
|  | 3 | 143 (60-195)  *13* |  |  |

**Table S3:** Blood pH, partial pressure of carbon dioxide (PaCO_2_), pressure of oxygen (PaO_2_), base excess (BE), bicarbonate (HCO_3_-), hemoglobin oxygen saturation (SaO_2_) and lactate (Lac) per group: Group 1, free-ranging (*n*=14), immobilized with *≈*2.5 µg/kg etorphine (high dose), *≈*2.5 µg/kg medetomidine, *≈*25 µg/kg midazolam and 1500-1700 IU hyaluronidase; Group 2, game-farmed (*n*=28), immobilized with *≈*2.5 µg/kg etorphine (high dose), *≈*2.5 µg/kg medetomidine, *≈*25 µg/kg midazolam and 1500-1700 IU hyaluronidase and Group 3, game-farmed animals (*n*=20) *≈*1 µg/kg etorphine (low dose), *≈*5 µg/kg medetomidine, *≈*25 µg/kg midazolam and 1700 IU hyaluronidase. Time sampling points: t_0_) at first handling, t_B_) after butorphanol administration and t_R_) at reversal. Data presented as Median (range) with *number of animals*.

| Variable (Unit) | Group | Time | | |
| --- | --- | --- | --- | --- |
|  |  | t0 | t_B_ | t_R_ |
| pH | 1 | 7.40 (7.31-7.51)  *14* | 7.44 (7.38-7.58)  *12* | 7.45 (7.35-7.61)  *9* |
|  | 2 | 7.32 (7.26-7.36)  *20* | 7.32 (7.29-7.35)  *20* | 7.32 (7.27-7.38)  *19* |
|  | 3 | 7.34 (7.28-7.38)  *18* |  | 7.33 (7.29-7.38)  *15* |
| PaCO_2_ (mmHg) | 1 | 45.2 (30.3-56.5)  *14* | 44.3 (16.9-53.2)  12 | 46 (34.0-67.5)  9 |
|  | 2 | 63.4 (55.9-78.2)  20 | 66.5 (47.6-73.6)  20 | 66.5 (56.6-74.6)  19 |
|  | 3 | 64.2 (55.7-70.3)  18 |  | 66.3 (55.0-75.4)  15 |
| PaO_2_ (mmHg) | 1 | 46.5 (24.0-81.0)  14 | 54.5 (21.0-118.0)  12 | 41.0 (23.0-60.0)  9 |
|  | 2 | 39.0 (14.0-55.0)  20 | 39.5 (23.0-54.0)  20 | 36.0 (30.0-63.0)  19 |
|  | 3 | 36.5 (25.0-53.0)  16 |  | 35.0 (27.0-45.0)  15 |
| HCO_3_- (mmol/L) | 1 | 28.2 (24.0-33.8)  14 | 29.2 (15.9-33.5)  12 | 32.2 (27.8-36.9)  9 |
|  | 2 | 33.5 (27.8-36.7)  20 | 33.56 ± 2.64  20 | 34.0 (29.9-39.1)  19 |
|  | 3 | 34.3 (32.0-37.8)  18 |  | 34.1 (31.7-38.0)  15 |
| BE (mmol/L) | 1 | 3.0 (0.0-9.0)  14 | 4.5 (-7.0-10.0)  12 | 8.0 (4.0-13.0)  9 |
|  | 2 | 7.5 (2.0-10.0)  20 | 8.0 (1.0-13.0)  20 | 8.0 (4.0-13.0)  19 |
|  | 3 | 8.0 (6.0-12.0)  18 |  | 8.0 (6.0-13.0)  15 |
| SaO_2_(%) | 1 | 82.5 (44.0-94.0)  14 | 89.0 (41.0-99.0)  12 | 75.0 (35.0-93.0)  9 |
|  | 2 | 67.0 (13.0-86.0)  20 | 66.5 (35.0-85.0)  20 | 63.0 (47.0-89.0)  19 |
|  | 3 | 63.5 (39.0-85.0)  18 |  | 60.0 (40.0-76.0)  15 |
| Lac (mmol/L) | 1 | 2.3 (0.8-5.4)  14 | 1.5 (0.5-4.1)  12 | 1.3 (0.6-3.1)  9 |
|  | 2 | 0.9 (0.4-5.4)  20 | 0.6 (0.4-3.9)  20 | 1.7 (0.6-3.2)  19 |
|  | 3 | 0.6 (0.3-0.9)  18 |  | 1.2 (0.3-2.7)  15 |

**Table S4:** Sodium (Na), potassium (K), chloride (Cl), ionized calcium (iCa), Glucose (Glu), urea (BUN), creatinine (Crea), and hematocrit (Hct) for all white rhinoceroses in this study and per group: Group 1, free-ranging (*n*=14), immobilized with *≈*2.5 µg/kg etorphine (high dose), *≈*2.5 µg/kg medetomidine, *≈*25 µg/kg midazolam and 1500-1700 IU hyaluronidase; Group 2, game-farmed (*n*=28), immobilized with *≈*2.5 µg/kg etorphine (high dose), *≈*2.5 µg/kg medetomidine, *≈*25 µg/kg midazolam and 1500-1700 IU hyaluronidase and Group 3, game-farmed animals (*n*=20) *≈*1 µg/kg etorphine (low dose), *≈*5 µg/kg medetomidine, *≈*25 µg/kg midazolam and 1700 IU hyaluronidase. Data presented as Median (range) with *n* (number of animals). The star indicates statistical significance (p < 0.05) with the reference (in Group 2 with Group 1 and in Group 3 with Group 2).

| **Variable (Unit)** | **Group** | | | |
| --- | --- | --- | --- | --- |
|  | **All groups**  **(*n* = 51)** | **1**  **(*n* =14)** | **2**  **(*n*=28)** | **3**  **(*n*=9)** |
| Na (mmol/L) | 132 (124-138) | 134 (125-137) | 132 (127-138) | 129* (124-131) |
| K (mmol/L) | 4.6 (3.8-5.6) | 5.2 (4.5-5.6) | 4.4* (3.8-5.6) | 4.4 (3.9-4.8) |
| Cl (mmol/L) | 92.0 (86.0-102.0) | 98.5 (96.0-102.0) | 92.0* (87.0-99.0) | 88.0* (86.0-91.0) |
| BUN (mg/dL) | 15 (8-19) | 14 (10-18) | 16 (8-19) | 16 (11-31) |
| Crea (mg/dL) | 1.3 (0.9-1.9) | 1.6 (1.3-1.9) | 1.3* (0.9-1.6) | 1.2 (1.0-1.4) |
| Glu (mg/dL) | 126 (86-201) | 136 (94-194) | 111 (86-188) | 150* (116-201) |
| iCa (mmol/L) | 1.51 (1.19-1.73) | 1.38 (1.19-1.51) | 1.60* (1.39-1.73) | 1.50* (1.41-1.58) |
| Hct (%PCV) | 42.0 (31.0-53.0) | 45.0 (38.0-53.0) | 42.5 (33.0-51.0) | 34.0* (31.0-50.0) |

**Table S5:** Coefficient estimates (standard errors) and p-values for time, group and position on pH, arterial partial pressure of carbon dioxide (PaCO_2_) and oxygen (PaO_2_), bicarbonate (HCO_3-_), base excess (BE), oxygen saturation (SaO_2_), lactate (Lac), mean- (MAP), systolic (SAP) and diastolic (DAP) non-invasive arterial blood pressure, heart rate (HR), respiratory rate (f_R_) and peripheral hemoglobin oxygen saturation (SpO_2_). Reference category: Group 1 (free-ranging, high etorphine dose), time t0, position lateral recumbency. The star indicates statistical significance (p < 0.05).

|  | **pH** | **PaCO_2_** | **PaO_2_** | **HCO_3-_** | **BE** | **SaO_2_** | **Lac** | **MAP** | **SAP** | **DAP** | **HR** | **f_R_** | **SpO_2_** |
| --- | --- | --- | --- | --- | --- | --- | --- | --- | --- | --- | --- | --- | --- |
| **Group 2** | -0.119^*^ | 22.343^*^ | -5.676 | 4.919^*^ | 3.007^*^ | -8.539 | -0.288 | 19.540^*^ | 22.301 | 16.989 | 0.527 | 0.864 | 5.141 |
|  | p = 0.000 | p = 0.000 | p = 0.216 | p = 0.00005 | p = 0.011 | p = 0.148 | p = 0.474 | p = 0.039 | p = 0.051 | p = 0.065 | p = 0.950 | p = 0.214 | p = 0.074 |
| **Time** | 0.008 | 0.431 | -0.371 | 0.673^*^ | 0.764^*^ | -0.048 | -0.0002 | 2.358 | 3.889 | 2.321 | 1.381^*^ | 0.119^*^ | -0.064 |
|  | p = 0.070 | p = 0.539 | p = 0.794 | p = 0.038 | p = 0.017 | p = 0.973 | p = 0.999 | p = 0.499 | p = 0.353 | p = 0.503 | p = 0.000 | p = 0.000 | p = 0.241 |
| **Position sternal** | -0.0002 | 1.497 | 3.435 | 0.800 | 0.746 | 4.031 | 0.310 | 29.833* | 27.620* | 31.140* | -3.105 | -1.223 | 6.973^*^ |
|  | p = 0.989 | p = 0.599 | p = 0.482 | p = 0.535 | p = 0.553 | p = 0.522 | p = 0.469 | p = 0.005 | p = 0.028 | p = 0.003 | p = 0.746 | p = 0.126 | p = 0.033 |
| **Position sternal to lateral** | -0.008 | -0.190 | 5.583 | -0.750 | -0.958 | 8.882 | -0.325 | 10.096 | -0.570 | 14.516 | -13.047 | -0.913 | 9.500 |
|  | p = 0.744 | p = 0.968 | p = 0.489 | p = 0.725 | p = 0.645 | p = 0.393 | p = 0.647 | p = 0.600 | p = 0.981 | p = 0.437 | p = 0.396 | p = 0.478 | p = 0.068 |
| **Constant** | 7.426^*^ | 41.202^*^ | 42.835^*^ | 27.067^*^ | 2.755 | 70.086^*^ | 1.546^*^ | 115.148^*^ | 159.492^*^ | 103.353^*^ | 52.955^*^ | 5.358^*^ | 68.743^*^ |
|  | p = 0.000 | p = 0.000 | p = 0.000 | p = 0.000 | p = 0.084 | p = 0.000 | p = 0.005 | p = 0.000 | p = 0.000 | p = 0.000 | p = 0.00001 | p = 0.00000 | p = 0.000 |
| **Observations** | 92 | 92 | 92 | 92 | 92 | 92 | 92 | 75 | 75 | 75 | 238 | 238 | 228 |
| **Log Likelihood** | 152.327 | -295.479 | -352.346 | -227.791 | -226.290 | -359.216 | -130.599 | -349.643 | -362.512 | -348.421 | -1,028.933 | -513.167 | -807.454 |
| **Akaike Inf. Crit.** | -290.654 | 604.958 | 718.691 | 469.581 | 466.579 | 732.431 | 275.198 | 713.285 | 739.024 | 710.841 | 2,071.867 | 1,040.335 | 1,628.907 |
|  |  |  |  |  |  |  |  |  |  |  |  |  |  |

**Table S6:** Coefficient estimates (standard errors) and p-values for time, group and position on pH, arterial partial pressure of carbon dioxide (PaCO_2_) and oxygen (PaO_2_), bicarbonate (HCO_3-_), base excess (BE), oxygen saturation (SaO_2_), lactate (Lac), mean- (MAP), systolic (SAP) and diastolic (DAP) non-invasive arterial blood pressure, heart rate (HR), respiratory rate (f_R_) and peripheral hemoglobin oxygen saturation (SpO2). Reference category: Group 2 (game-farmed, high etorphine dose), time t0, position lateral recumbency. The star indicates statistical significance (p < 0.05).

|  | **pH** | **PaCO_2_** | **PaO_2_** | **HCO_3-_** | **BE** | **SaO_2_** | **Lac** | **MAP** | **SAP** | **DAP** | **HR** | **f_R_** | **SpO_2_** |
| --- | --- | --- | --- | --- | --- | --- | --- | --- | --- | --- | --- | --- | --- |
| **Group 3** | 0.010 | -0.283 | -1.907 | 0.625 | 0.781 | -1.500 | -0.406 | 0.416 | 2.299 | 3.315 | -20.903^*^ | -1.478^*^ | 1.488 |
|  | p = 0.090 | p = 0.843 | p = 0.373 | p = 0.325 | p = 0.235 | p = 0.671 | p = 0.107 | p = 0.964 | p = 0.832 | p = 0.720 | p = 0.0003 | p = 0.004 | p = 0.463 |
| **Time** | -0.003 | 0.712 | -0.160 | 0.102 | 0.062 | -0.287 | 0.292^*^ | 4.154 | 6.704 | 3.637 | 1.358^*^ | 0.126^*^ | -0.076 |
|  | p = 0.300 | p = 0.061 | p = 0.822 | p = 0.427 | p = 0.674 | p = 0.804 | p = 0.002 | p = 0.269 | p = 0.097 | p = 0.349 | p = 0.000 | p = 0.000 | p = 0.093 |
| **Position sternal** | -0.003 | 1.395 | 0.868 | 0.545 | 0.448 | 0.152 | -0.092 | 30.209* | 31.482* | 31.589* | -4.074 | -0.972 | 6.062^*^ |
|  | p = 0.623 | p = 0.326 | p = 0.684 | p = 0.392 | p = 0.496 | p = 0.966 | p = 0.712 | p = 0.0002 | p = 0.002 | p = 0.0001 | p = 0.490 | p = 0.060 | p = 0.004 |
| **Position sternal to lateral** | -0.010 | -0.241 | 4.284 | -0.855 | -1.082 | 6.939 | -0.534 | 10.368 | 2.163 | 14.813 | -13.802 | -0.719 | 8.890^*^ |
|  | p = 0.464 | p = 0.940 | p = 0.357 | p = 0.552 | p = 0.465 | p = 0.367 | p = 0.324 | p = 0.562 | p = 0.923 | p = 0.403 | p = 0.263 | p = 0.505 | p = 0.040 |
| **Constant** | 7.329^*^ | 63.033^*^ | 38.035^*^ | 33.234^*^ | 7.291^*^ | 63.968^*^ | 0.884^*^ | 130.825^*^ | 173.429^*^ | 117.413^*^ | 54.444^*^ | 5.929^*^ | 74.665^*^ |
|  | p = 0.000 | p = 0.000 | p = 0.000 | p = 0.000 | p = 0.000 | p = 0.000 | p = 0.002 | p = 0.000 | p = 0.000 | p = 0.000 | p = 0.000 | p = 0.000 | p = 0.000 |
| **Observations** | 92 | 92 | 90 | 92 | 92 | 90 | 90 | 71 | 71 | 71 | 258 | 258 | 253 |
| **Log Likelihood** | 194.898 | -256.071 | -295.301 | -171.476 | -179.950 | -336.877 | -117.471 | -324.855 | -334.770 | -325.704 | -1,120.608 | -557.868 | -860.572 |
| **Akaike Inf. Crit.** | -375.797 | 526.143 | 604.602 | 356.952 | 373.900 | 687.753 | 248.942 | 663.710 | 683.540 | 665.408 | 2,255.216 | 1,129.735 | 1,735.143 |
